# Supplementary material for: Multilevel Comparison of Indian Naja Venoms and Their Cross-Reactivity with Indian Polyvalent Antivenoms
Source: Toxins (Basel). 2023 Apr 1;15(4):258. doi: 10.3390/toxins15040258 (PMC10142961; doi:10.3390/toxins15040258)
Supplement: Supplementary file 1 [file toxins-15-00258-s001.zip › Supplementary table S1-Compiled proteomics of naja spp-V4.pdf]

Supplementary Table S1: Toxins were assigned to peptides sequenced from MS/MS spectra using Proteome Discoverer. Protein were identified by comparing fragment masses against Elapidae database in NCBI. Only proteins with a SEQUEST score of 1.0 or higher are listed. Toxin abundance was calculated based on its mean spectral intensity (MSI) relative to the total spectral intensity of all proteins detected.

Sample ID	Accession	Description	Toxin family	Coverage [%]	MW [kDa]	Score Sequest HT	Percent abundance	Sequence
Naja naja (Arunachal Pradesh)	AAB18383.1	cardiotoxin 3a [*Naja atra*]	Cardiotoxin	69	9.1	69.39	0.371	RGCIDVCPK<br>MFMVATPK<br>YVCCNTDR
	BAA36404.1	phospholipase A2 [*Naja kaouthia*]	PLA2	65	16	49.49	0.304	NMIQCTVPSR<br>ISGCWPYFK<br>TYSYECSQGTLTCK
	AAB25732.1	cardiotoxin isoform 1, cytotoxin isoform 1, CTX-1 [*Naja naja* =Formosan cobra, ssp. atra, venom, Peptide, 60 aa]	Cardiotoxin	77	6.7	47.82	0.022	MFMMSDLTIPVK<br>MFMMSDLTIPVKR<br>GCIDVCPKSNLLVK
	AAF00693.1	cobrin precursor [*Naja naja*]	SVMP	23	67.6	39.93	0.017	NSMICNCSISPR<br>MVAITMAHEMGHNLGMNHDK<br>RNSMICNCSISPR
	P14541.1	RecName: Full=Cytotoxin homolog; AltName: Full=Cytotoxin-like basic protein; Short=CLBP	3FTx	52	7	39.72	0.046	YVCCSTDK<br>YVCCSTDKCN<br>LKCHNTQLPFIYK
	BAU24670.1	cytotoxin 13, partial [*Naja naja*]	3FTx	67	7.9	36.89	0.071	LIPLAYKTCPAGK<br>MFMVSNK
	3PVM	D Chain D, Cobra Venom Factor	CVF	11	184.4	36	0.003	VNDDYLIWGSR<br>VAVIIYLNK<br>THQYISQR<br>LNQDITVTASGDGK<br>INYENALLAR
	D3TTC2.1	RecName: Full=Zinc metalloproteinase-disintegrin-like atragin; AltName: Full=Snake venom metalloproteinase; Short=SVMP; Flags: Precursor	SVMP	20	69.1	32.34	0.003	NGLPCQNNQGYCYNGK<br>DPNYGMVEPGTK<br>RTKPAYQFSSCSVR
	5GZ4	A Chain A, Snake Venom Phosphodiesterase (pde)	PDE	17	94.6	27.9	0.003	NPFYNPSPAK<br>MANVLCSCSEDCLTKK<br>YCLLHQTK
	ADF43026.1	metalloproteinase atrase A [*Naja atra*]	SVMP	18	68.2	27.79	0.008	NGHPCQNNQGYCYNGK<br>VYEMVNYLNTK<br>RTILMASTMAHELGHNMGIHHDK
	P29180.1	RecName: Full=Weak neurotoxin 6	3FTx	78	7.6	26.19	0.025	LTCLICPEK<br>VHTCLNGEK<br>VHTCLNGEKICFK
	5H7W	A Chain A, venom 5'-nucleotidase	5'-nucleotidase	28	58.2	25.25	0.002	NVKFPILSANIRPK<br>QVPVVQAYAFGK<br>VVSLNVLCTECR
	1V6P	B Chain B, Cobrotoxin	3FTx	63	7	20.88	0.055	NGIEINCCTTDR<br>LECHNQQSSQTPTTTGCSGGETNCYK<br>LECHNQQSSQTPTTTGCSGGETNCYKK
	Q7T1K6.1	RecName: Full=Cysteine-rich venom protein natrin-1; AltName: Full=Cysteine-rich venom protein 1; AltName: Full=NA-CRVP1; AltName: Full=Protein G2a; Flags: Precursor	CRISP	28	26.9	19.52	0.009	QKEIVDLHNSLR<br>QSSCQDDWIK<br>LTNCDSLLK
	5Z2G	A Chain A, L-amino acid oxidase	LAAO	17	57.9	19.3	0.002	REIQALCYPSIKK<br>RIYFEPPLPPK<br>VTLLEASER
	AFJ59923.1	OVF precursor protein [*Ophiophagus hannah*]	Complement C3	5	183.8	17.72	0.001	QLDIFVHDFPR<br>IWDTIEK<br>TDTEEQILVEAHGDNTPK
	AAM51550.1	mocarhagin 1 [*Naja mossambica*]	SVMP	8	68.1	17.31	0.002	NDCDFPELCTGR<br>VYEMVNALNTMYR<br>SVAVVQDHSK
	P01400.1	RecName: Full=Weak toxin S4C11	3FTx	74	7.4	17.1	0.006	EIVECCSTDKCNH<br>FYEGNLLGK<br>GCAATCPEAKPR
	Q9DEQ3.1	RecName: Full=Neurotoxin homolog NL1; Flags: Precursor	3FTx	29	10	14.03	0.006	ISLADGNDVR<br>RGCTFTCPELRPTGK<br>GCTFTCPELRPTGK
	P59276.1	RecName: Full=Cobrotoxin-c; Short=CBT-c; AltName: Full=Short neurotoxin II; Short=NT2	3FTx	70	6.9	13.29	0.035	VKPGVNLNCCR<br>TCSGETNCYKK<br>KWWSDHR
	AAP20603.1	cysteine-rich venom protein [*Naja atra*]	CRISP	18	26.2	12.15	0.001	VIQSWYDENKK<br>CSFAHSPPHLR<br>CAASCFCR
	ETE68810.1	Glutathione peroxidase 3, partial [*Ophiophagus hannah*]	Peroxidase	18	29.6	11.66	0.001	QEPGQNSEILQGIK<br>TNVSTVKNDIIR<br>FLVNPQGKPVMR
	P49122.1	RecName: Full=Cytotoxin 7; AltName: Full=Cardiotoxin-7; Short=CTX7; Short=Ctx-7; AltName: Full=Cardiotoxin-like basic protein 2; Short=CLBP2; Flags: Precursor	3FTx	35	9.1	11.6	0.005	FPLKFPVK<br>ATLKFPLKFPVK<br>CHNTQLPFIYNTCPEGK
	P82463.1	RecName: Full=Muscarinic toxin-like protein 2; Short=MTLP-2	3FTx	69	7.3	5.99	0.001	TRGCAATCPIAENR

								DVIECCSTDK<br>GCAATCPIAENR
Naja Kaouthia (Mizoram)	AAB18384.2	cardiotoxin 3b [*Naja atra*]	Cardiotoxin	72	9	67.81	0.612	MFMVATPKVPVKR<br>LVPLFYKTCPAGK<br>YVCCNTDR
	3PVM	D Chain D, Cobra Venom Factor	CVF	20	184.4	64.78	0.010	LNQDITVTASGDGK<br>INYENALLAR<br>IPIIDGDGK
	BAA36404.1	phospholipase A2 [*Naja kaouthia*]	PLA2	40	16	45.52	0.233	NMIQCTVPSR<br>TYSYECSQGTLTCK<br>CCQVHDNCYNEAEK
	Q9PST3.1	RecName: Full=Cytotoxin 2b; AltName: Full=Cardiotoxin-2b; Short=CTX-2b; Short=Ctx2b; Flags: Precursor	3FTx	93	9	36.04	0.000	MYMVATPK<br>GCIDVCPKSSLLVK<br>LTLVVVTTVCLDLGYTLKCNKLVPLFYK
	3HRZ	A Chain A, Cobra Venom Factor	CVF	16	69.5	27.62	0.001	ALYTLITPAVLR<br>VGLVAVDK<br>YFTYLILNK
	P01446.1	RecName: Full=Cytotoxin 3; Short=CX3; AltName: Full=Toxin CM-7	3FTx	90	6.7	27.28	0.033	MFMVSNK<br>LKCNKLIPIAYK<br>LIPLAYKTCPAGK
	P14541.1	RecName: Full=Cytotoxin homolog; AltName: Full=Cytotoxin-like basic protein; Short=CLBP	3FTx	48	7	25.08	0.010	LKCHNTQLPFIYK<br>YVCCSTDK<br>CHNTQLPFIYK
	P29180.1	RecName: Full=Weak neurotoxin 6	3FTx	69	7.6	24.9	0.005	LTCLICPEKYCNK<br>VHTCLNGEK<br>EIVQCCSTDK
	AFJ59923.1	OVF precursor protein [*Ophiophagus hannah*]	CVF	5	183.8	24.72	0.000	QLDIFVHDFPR<br>TDTEEQILVEAHGDNTPK<br>FYHPDKGTGLINK
	P49122.1	RecName: Full=Cytotoxin 7; AltName: Full=Cardiotoxin-7; Short=CTX7; Short=Ctx-7; AltName: Full=Cardiotoxin-like basic protein 2; Short=CLBP2; Flags: Precursor	3FTx	37	9.1	24.52	0.009	FPLKFPVK<br>ATLKFPLKFPVK<br>CHNTQLPFIYNTCPEGK
	AAF00693.1	cobrin precursor [*Naja naja*]	SVMP	17	67.6	23.91	0.002	MVAITMAHEMGHNLGMNHDK<br>NSMICNCSISPR<br>LQHEAQCDSEECCEK
	PSNJ2K	phospholipase A2 (EC 3.1.1.4) II - monocled cobra	PLA2	34	13.4	23.57	0.017	NGNNACAAAVCDCDR<br>GGSGTPVDDLDR
	1V6P	B Chain B, Cobrotoxin	3FTx	68	7	20.98	0.020	NGIEINCCTTDR<br>LECHNQQSSQTPTTTGCSGGETNCYK<br>NGIEINCCTTDRCNN
	1XTA	A Chain A, Crystal Structure Of Natrin, A Snake Venom Crisp From Taiwan Cobra (*Naja atra*)	CRISP	36	24.9	19.94	0.009	MEWYPEAASNAER<br>LTNCDSLLK<br>NVDFNSESTR
	BAU24674.1	cytotoxin 15, partial [*Naja naja*]	3FTx	60	8	19.24	0.004	MYMVSNK
	D3TTC2.1	RecName: Full=Zinc metalloproteinase-disintegrin-like atragin; AltName: Full=Snake venom metalloproteinase; Short=SVMP; Flags: Precursor	SVMP	16	69.1	18.38	0.007	NGLPCQNNQGYCYNGK<br>DPNYGMVEPGTK
	AAB25732.1	cardiotoxin isoform 1, cytotoxin isoform 1, CTX-1 [*Naja naja* =Formosan cobra, ssp. atra, venom, Peptide, 60 aa]	Cardiotoxin	80	6.7	18.18	0.000	MFMMSDLTIPVKR<br>TCPAGKNLCYK
	BAU24666.1	cytotoxin 11, partial [*Naja naja*]	3FTx	51	8	16.69	0.000	MFMVSDLTIPVKR<br>GCIDVCPKNSLLVK
	ADF43026.1	metalloproteinase atrase A [*Naja atra*]	SVMP	13	68.2	15.37	0.006	VYEMVNYLNTK<br>TRVYEMVNYLNTK
	5H7W	A Chain A, venom 5'-nucleotidase	5'-nucleotidase	16	58.2	14.35	0.001	IIALGHSGFMEDCR<br>VPTYVPLEMEK
	JAS05143.1	ecto-5'-nucleotidase [*Micrurus tener*]	5'-nucleotidase	12	63	13.75	0.000	LTILHTNDVHAR<br>VPTYVPLQMEK<br>QVPVVQAYAFGK
	P82942.1	RecName: Full=Hemorrhagic metalloproteinase-disintegrin-like kaouthiagin; AltName: Full=Snake venom metalloproteinase; Short=SVMP	SVMP	24	44.5	10.57	0.007	NGHPCQNNQGYCYNGK<br>QTVLLPR
	5GZ4	A Chain A, Snake Venom Phosphodiesterase (pde)	PDE	10	94.6	10.39	0.001	NPFYNPSPAK<br>RPDFSTLYIEEPDTTGHK<br>SMEAIFLAHGPGFK
	AAP20603.1	cysteine-rich venom protein [*Naja atra*]	CRISP	18	26.2	9.3	0.000	VIQSWYDENKK<br>CAASCFCR<br>HHNVFSNCQSLAK
	ETE68810.1	Glutathione peroxidase 3, partial [*Ophiophagus hannah*]	Peroxidase	14	29.6	8.74	0.001	TNVSTVKNDIIR<br>FLVNPQGKPVMR<br>QEPGQNSEILQGIK
	Q9DEQ3.1	RecName: Full=Neurotoxin homolog NL1; Flags: Precursor	3FTx	29	10	7.76	0.001	ISLADGNDVR<br>RGCTFTCPELRPTGK<br>GCTFTCPELRPTGK

	1CDT	A Chain A, CARDIOTOXIN VII4	Cardiotoxin	45	6.7	7.48	0.000	CNKLIPLAYK<br>GCINVCPK
	3K7N	A Chain A, Structures Of Two Elapid Snake Venom Metalloproteases With Distinct Activities Highlight The Disulfide Patterns In The D Domain Of Adamalysin Family Proteins	SVMP	11	44.2	6.61	0.000	YYNNDKPAIK<br>VYEMINAVNTK<br>TAPAFQFSSCSIR
	P82885.1	RecName: Full=Thaicobrin	3FTx	37	12	6.29	0.001	EWAVGLAGK<br>FDGSPCVLGSPGFR<br>TVENVGVSQVAPDNPER
	P82463.1	RecName: Full=Muscarinic toxin-like protein 2; Short=MTLP-2	3FTx	37	7.3	5.61	0.001	TRGCAATCPIAENR<br>GCAATCPIAENR<br>GCAATCPIAENRDVIECCSTDK
	P01427.1	RecName: Full=Short neurotoxin 1; AltName: Full=Neurotoxin II; Short=NT II; Short=NTII; Short=NTX II; AltName: Full=Neurotoxin alpha	3FTx	36	6.9	4.98	0.008	VKPGVNLNCCR<br>TCSGETNCYK<br>TCSGETNCYKK
	AAM51550.1	mocarhagin 1 [*Naja mossambica*]	SVMP	4	68.1	3.86	0.000	AAKNDCDFPELCTGR<br>SVAVVQDHSK<br>NDCDFPELCTGR
	5Z2G	A Chain A, L-amino acid oxidase	LAAO	4	57.9	1.71	0.000	SASQLYQESLR<br>IFLTCSK<br>SASQLYQESLRK
Naja Kaouthia (West Bengal)	BAU24666.1	cytotoxin 11, partial [*Naja naja*]	3FTx	51	8	100.46	0.351	MFMVSDLTIPVK<br>YVCCNTDR<br>GCIDVCPK
	3PVM	D Chain D, Cobra Venom Factor	CVF	23	184.4	82.57	0.014	AAFLECCR<br>QLDIFVHDFPR<br>QNQYVVVQVTGPQVR
	BAA36404.1	phospholipase A2 [*Naja kaouthia*]	PLA2	65	16	46.34	0.275	NMIQCTVPSR<br>TYSYECSQGTLTCK<br>ISGCWPYFK
	AFJ59923.1	OVF precursor protein [*Ophiophagus hannah*]	CVF	11	183.8	42.58	0.002	VSHSEDECLQFK<br>TDTEEQILVEAHGDNTPK<br>KLDDKVPDTEIETK
	3HRZ	A Chain A, Cobra Venom Factor	CVF	16	69.5	32.56	0.001	ALYTLITPAVLR<br>IWDTIEK
	CAM34525.2	phospholipase A2, partial [*Naja atra*]	PLA2	65	12.3	31.75	0.002	MIQCTVPSR
	1V6P	B Chain B, Cobrotoxin	3FTx	63	7	30.82	0.051	NGIEINCCTTDR<br>LECHNQQSSQTPTTTGCSGGETNCYK
	AAF00693.1	cobrin precursor [*Naja naja*]	SVMP	18	67.6	27.35	0.007	NSMICNCSISPR<br>RTKPAYQFSSCSVR<br>MVAITMAHEMGHNLGMNHDK
	1KXI	B Chain B, Structure Of Cytotoxin Homolog Precursor	3FTx	37	7	26.58	0.013	FPLKFPVKR<br>CHNTQLPFIYK<br>LKCHNTQLPFIYK
	D3TTC2.1	RecName: Full=Zinc metalloproteinase-disintegrin-like atragin; AltName: Full=Snake venom metalloproteinase; Short=SVMP; Flags: Precursor	SVMP	13	69.1	26.15	0.001	ATLNLFGEWR<br>DPNYGMVEPGTK<br>TKPAYQFSSCSVR
	5H7W	A Chain A, venom 5'-nucleotidase	5'-Nucleotidase	23	58.2	25.85	0.002	QVPVVQAYAFGK<br>VGIIGYTTK<br>VPTYVPLEMEK
	5Z2G	A Chain A, L-amino acid oxidase	LAAO	13	57.9	23.14	0.001	REIQALCYPSIK<br>SASQLYQESLR<br>STTDLPSR
	AAB18383.1	cardiotoxin 3a [*Naja atra*]	Cardiotoxin	48	9.1	21.14	0.051	MFMVATPK
	P25668.1	RecName: Full=Long neurotoxin 1; AltName: Full=Toxin A	3FTx	63	7.8	20.29	0.007	RVDLGCAATCPTVR<br>TWCDGFCSIR<br>VDLGCAATCPTVR
	4AEA	A Chain A, Long Neurotoxin 1	3FTx	75	7.8	19.6	0.001	VDLGCAATCPTVK<br>TGVDIQCCSTDNCNPFPTR<br>CFITPDITSK
	P01400.1	RecName: Full=Weak toxin S4C11	3FTx	69	7.4	19.46	0.008	LTCLICPEK<br>EIVECCSTDK<br>FYEGNLLGK
	5GZ4	A Chain A, Snake Venom Phosphodiesterase (pde)	PDE	8	94.6	17.88	0.001	NPFYNPSPAK<br>QPLSETLR<br>SMEAIFLAHGPGFK
	P82463.1	RecName: Full=Muscarinic toxin-like protein 2; Short=MTLP-2	3FTx	83	7.3	16.42	0.006	SIFGVTTEDCPDGQNLCFKR<br>WHMLVPGR<br>TRGCAATCPIAENR
	ETE68810.1	Glutathione peroxidase 3, partial [*Ophiophagus hannah*]	Peroxidase	17	29.6	14.83	0.002	QEPGQNSEILQGIK<br>TNVSTVKNDIIR<br>FLVNPQGKPVMR
	P59276.1	RecName: Full=Cobrotoxin-c; Short=CBT-c; AltName: Full=Short neurotoxin II; Short=NT2	3FTx	80	6.9	14.58	0.056	VKPGVNLNCCR<br>WWSDHRGTIIER<br>TCSGETNCYKK
	AAK49439.1	cardiotoxin [*Naja sputatrix*]	Cardiotoxin	47	9	13.65	0.144	MYMVATPKVPVKR

								LVPLFYK
	1XTA	A Chain A, Crystal Structure Of Natrin, A Snake Venom Crisp From Taiwan Cobra (*Naja atra*)	CRISP	31	24.9	12.77	0.003	NVDFNSESTR<br>QKEIVDLHNSLR<br>LTNCDSLLK
	AAX86641.1	venom factor [*Austrelaps superbus*]	CVF	4	184.8	11.4	0.001	GNANSLNQIK<br>YFTYLILTK
	AAB24494.1	Vc-5=cytotoxin [Naja oxiana=snakes, venom, Peptide, 60 aa]	3FTx	80	6.7	83.73	0.05061	MFMVAAPHVPVK<br>YVCCNTDR<br>TCPAGKNLCYK
	P25669.1	RecName: Full=Long neurotoxin 2; AltName: Full=Toxin B	3FTx	66	7.8	83.65	0.04904	RVDLGCAATCPTVR<br>VDLGCAATCPTVR<br>TWCDGFCSSR
	P25668.1	RecName: Full=Long neurotoxin 1; AltName: Full=Toxin A	3FTx	66	7.8	75.5	0.21518	DCPNGHVCYTK<br>CFITPDITSK
	P25672.1	RecName: Full=Long neurotoxin 4; AltName: Full=Toxin D	3FTx	63	7.9	46.98	0.03158	VDLGCAATCPTVK<br>TWCDGFCR<br>GERVDLGCAATCPTVK
	1KXI	B Chain B, Structure Of Cytotoxin Homolog Precursor	3FTx	53	7	43.46	0.04113	YVCCSTDK<br>FPLKFPVK<br>CHNTQLPFIYK
	3K7L	A Chain A,Snake Venom Metalloproteases	SVMP	32	47.7	40.48	0.02349	NGLPCQNNQGYCYNGK<br>TNTPEQDRYLQAK<br>DPNYGMVEPGTK
	CAA45372.1	phospholipase a2 [*Naja naja*]	PLA2	53	13.5	40.19	0.01144	ISGCWPYFK<br>TYSYECSQGTLTCK<br>CCQVHDNCYNEAEK
	AAF00693.1	cobrin precursor [*Naja naja*]	SVMP	19	67.6	36.38	0.00237	NSMICNCSISPR<br>RTKPAYQFSSCSVR<br>TSAAVVQDYSK
	PSNJ3K	Phospholipase A2 (EC 3.1.1.4) III - monocled cobra	PLA2	67	13.3	36.1	0.23539	NMIQCTVPSR<br>CCQVHDNCYDEAEK
	5H7W	A Chain A, venom 5'-nucleotidase	5'-nucleotidase	22	58.2	33.55	0.00231	NVKFPILSANIRPK<br>QVPVVQAYAFGK<br>SIQEDPAVK
	P82942.1	RecName: Full=Hemorrhagic metalloproteinase-disintegrin-like kaouthiagin; AltName: Full=Snake venom metalloproteinase; Short=SVMP	SVMP	30	44.5	33.2	0.01624	QTVLLPR<br>RTAPAFQFSSCSIR<br>NGHPCQNNQGYCYNGK
	5Z2G	A Chain A, L-amino acid oxidase	LAAO	23	57.9	33.2	0.00453	REIQALCYPSIK<br>RIYFEPPLPPK<br>SASQLYQESLR
	3PVM	D Chain D, Cobra Venom Factor	CVF	13	184.4	31.53	0.00210	QLDIFVHDFPR<br>VGLVAVDK<br>VNDDYLIWGSR
	CAM34525.2	phospholipase A2, partial [*Naja atra*]	PLA2	52	12.3	29.3	0.00916	MIQCTVPSR
	JAS05143.1	ecto-5'-nucleotidase [*Micrurus tener*]	5'-nucleotidase	16	63	27.54	0.00024	LTILHTNDVHAR<br>VPTYVPLQMEK
	2WQ5	A Chain A, Phospholipase A2, Acidic	PLA2	34	13.3	26.39	0.01052	GGSGTPVDDLDR<br>GGNNACAASVCDCDR
	5GZ4	A Chain A, Snake Venom Phosphodiesterase (pde)	PDE	15	94.6	25.9	0.00252	NPFYNPSPAK<br>MANVLCSCSEDCLTK<br>YCLLHQTK
	3K7N	A Chain A, Structures Of Two Elapid Snake Venom Metalloproteases With Distinct Activities Highlight The Disulfide Patterns In The D Domain Of Adamalysin Family Proteins	SVMP	18	44.2	25.87	0.00142	RNDNAQLLTGIDFNGNTVGR<br>VYEMINAVNTK
Naja naja (Maharashtra): Sample A	P86540.2	RecName: Full=Cytotoxin 8; Short=CTX8	3FTx	75	6.8	25.53	0.00574	NSLLVKYECCNTDR<br>MYMVSDKTVPVKR<br>YECCNTDR
	BAU24674.1	cytotoxin 15, partial [*Naja naja*]	3FTx	64	8	24.7	0.15770	MYMVSNK<br>MYMVSNKTVPVKR
	AAK49439.1	cardiotoxin [*Naja sputatrix*]	Cardiotoxin	52	9	23.86	0.06060	MYMVATPK
	P86538.2	Cytotoxin 2a; Short=CTX2a; AltName: Full=Cytotoxin 2; Short=CTX2	3FTx	58	6.7	23.33	0.00011	MFMVSDLTIPVKR<br>GCIDVCPK
	ADF43026.1	metalloproteinase atrase A [*Naja atra*]	SVMP	14	68.2	23.21	0.01360	VYEMVNYLNTK<br>TRVYEMVNYLNTK
	ETE68810.1	Glutathione peroxidase 3, partial [*Ophiophagus hannah*]	GPx	18	29.6	21.36	0.00173	QEPGQNSEILQGIK<br>TNVSTVKNDIIR<br>FLVNPQGKPVMR
	O73858.1	RecName: Full=Cytotoxin 6; AltName: Full=Cardiotoxin-6; Short=CTX-6; Short=Ctx6; Flags: Precursor	3FTx	76	7	18.72	0.02027	MFMVSNK
	P20229.1	RecName: Full=Kunitz-type serine protease inhibitor; AltName: Full=Venom trypsin inhibitor	KUN	58	6.4	17.97	0.01576	RPGFCELPAAK<br>FIYGGCGGNANR<br>AHKPAFYYNK

3HRZ	A Chain A, Cobra Venom Factor	CVF	12	69.5	16.37	0.00027	ALYTLITPAVLR<br>DTCMGTLVVK
1XTA	A Chain A, Crystal Structure Of Natrin, A Snake Venom Crisp From Taiwan Cobra (*Naja atra*)	CRISP	32	24.9	16.15	0.00432	NVDFNSESTR<br>QKEIVDLHNSLR<br>RVSPTASNMLK
AFJ59923.1	OVF precursor protein [*Ophiophagus hannah*]	CVF	5	183.8	13.87	0.00142	TDTEEQILVEAHGDNTPK<br>KLDDKVPDTEIETK
AAM51550.1	mocarhagin 1 [*Naja mossambica*]	SVMP	4	68.1	11.08	0.00069	NDCDFPELCTGR<br>SVAVVQDHSK<br>AAKNDCDFPELCTGR
AAP20603.1	cysteine-rich venom protein [*Naja atra*]	CRISP	19	26.2	10.29	0.00099	NMLQMEWNSNAAQNAK<br>QNACQTEWMK<br>CSFAHSPPHLR
P82463.1	RecName: Full=Muscarinic toxin-like protein 2; Short=MTLP-2	3FTx	80	7.3	10.24	0.00140	WHMIVPGR<br>SIFGVTTEDCPDGQNLCFKR<br>TRGCAATCPIAENR
P01460.1	RecName: Full=Cytotoxin 8; AltName: Full=Toxin CM-7	3FTx	30	6.8	9.34	0.00133	YVCCNTNK<br>YVCCNTNKCN
JAI08992.1	Metalloproteinase (type III) 1 [*Micrurus fulvius*]	SVMP	6	69	7.6	0.00022	CPTDSFQR<br>CGDGMVCSNR
XP_026544671.1	tissue-type plasminogen activator, partial [*Notechis scutatus*]	CRISP	6	60.6	5.97	0.00056	SSKPWCHVLK<br>TLDNDIALLK<br>TVTENMLCAGDTR
P29180.1	RecName: Full=Weak neurotoxin 6	3FTx	62	7.6	5.69	0.00126	VHTCLNGEK<br>GCADTCPVR<br>LTCLICPEK
P82464.1	RecName: Full=Muscarinic toxin-like protein 3; Short=MTLP-3	3FTx	60	7.6	4.89	0.00265	ISLADGNDVR<br>TICYNHLTR<br>GCTFTCPELRPTGIYVYCCR
ABQ01138.1	scutatease-1 [*Notechis scutatus*]	SVMP	7	68	2.54	0.00008	AAKDDCDLPESCTGQSAECPMDSFQR
AAB19290.1	miscellaneous type neurotoxin [*Naja naja* =cobra, ssp. naja, Peptide, 65 aa]	3FTx	35	7.6	2.26	0.00002	GCAATCPEAKPR<br>EIVQCCSTDK<br>RGCAATCPEAKPR
AAB24494.1	Vc-5=cytotoxin [Naja oxiana=snakes, venom, Peptide, 60 aa]	3FTx	90	6.7	132.85	0.12589	RGCIDVCPK<br>MFMVAAPHVPVKR<br>YVCCNTDR
P25668.1	RecName: Full=Long neurotoxin 1; AltName: Full=Toxin A	3FTx	66	7.8	64.46	0.19629	VDLGCAATCPTVR<br>TWCDGFCSIR<br>RVDLGCAATCPTVR
P25669.1	RecName: Full=Long neurotoxin 2; AltName: Full=Toxin B	3FTx	66	7.8	64.22	0.05047	TWCDGFCSSR
1KXI	B Chain B, Structure Of Cytotoxin Homolog Precursor	3FTx	76	7	59.01	0.04151	LKCHNTQLPFIYK<br>YVCCSTDK<br>FPLKFPVK
P60309.1	RecName: Full=Cytotoxin SP15d	3FTx	72	6.6	57.41	0.03984	RGCINVCPK<br>MFMVAAPKVPVKR<br>YVCCNTDK
BAU24670.1	cytotoxin 13, partial [*Naja naja*]	3FTx	78	7.9	56.25	0.04046	LIPLAYKTCPAGK<br>MFMVSNKTVPVKR
P86540.2	RecName: Full=Cytotoxin 8; Short=CTX8	3FTx	97	6.8	49.41	0.00512	NSLLVKYECCNTDR<br>MYMVSDKTVPVKR
D3TTC2.1	RecName: Full=Zinc metalloproteinase-disintegrin-like atragin; AltName: Full=Snake venom metalloproteinase; Short=SVMP; Flags: Precursor	SVMP	20	69.1	44.23	0.00524	NGLPCQNNQGYCYNGK<br>NSMICNCSISPR<br>RTKPAYQFSSCSVR
4AEA	A Chain A, Long Neurotoxin 1	3FTx	76	7.8	43.49	0.05657	VDLGCAATCPTVK<br>TGVDIQCCSTDNCNPFPTR<br>CFITPDITSKDCPNGHVCYTK
3K7N	A Chain A, Structures Of Two Elapid Snake Venom Metalloproteases With Distinct Activities Highlight The Disulfide Patterns In The D Domain Of Adamalysin Family Proteins	SVMP	30	44.2	41.85	0.01349	NDNAQLLTGIDFNGNTVGR<br>RNDNAQLLTGIDFNGNTVGR<br>MEDGTKIPCAAK
CAA45372.1	phospholipase a2 [*Naja naja*]	PLA2	83	13.5	41.53	0.15943	ISGCWPYFK<br>TYSYECSQGTLTCKGDNNACAASVCDCDR<br>SWWDFADYGCYCGR
P01447.1	RecName: Full=Cytotoxin 1; AltName: Full=Cobramine-A; AltName: Full=Cytotoxin I; AltName: Full=Cytotoxin XI; AltName: Full=Cytotoxin-like basic protein; Short=CLBP	3FTx	97	6.8	41.46	0.13979	NSLVLKYECCNTDRCN<br>MYMVSNKTVPVKR
AAF00693.1	cobrin precursor [*Naja naja*]	SVMP	23	67.6	36.3	0.02213	MVAITMAHEMGHNLGMNHDK<br>TSAAVVQDYSK<br>DPSYGMVEPGTK<br>ATLDLFGEWR
5Z2G	A Chain A, L-amino acid oxidase	LAAO	30	57.9	35.08	0.00304	REIQALCYPSIKK<br>RIYFEPPLPPKK<br>IHFAGEYTGR

Naja naja (Maharashtra): Sample B	PSNJ3K	phospholipase A2 (EC 3.1.1.4) III - monocled cobra	PLA2	79	13.3	34.73	0.02301	NMIQCTVPSR<br>SWWNFADYGCYCGR
	CAM34525.2	phospholipase A2, partial [*Naja atra*]	PLA2	52	12.3	33.85	0.00024	MIQCTVPSR<br>TYSYECSQGTLTCK
	P07525.1	1007132A cytotoxin D1	3FTx	85	6.8	33.3	0.00009	CNKLVPLFYK<br>LVPLFYK
	P62394.1	RecName: Full=Cytotoxin 11; AltName: Full=Toxin CM-12	3FTx	37	6.8	32.55	0.00014	CHNTQLPFIYK<br>VVCCSTDKCN
	5H7W	A Chain A, venom 5'-nucleotidase	5'-nucleotidase	20	58.2	32.16	0.00230	NVKFPILSANIRPK<br>QVPVVQAYAFGK<br>IIALGHSGFMEDCR
	P25672.1	RecName: Full=Long neurotoxin 4; AltName: Full=Toxin D	3FTx	52	7.9	32.03	0.00035	GERVDLGCAATCPTVK<br>DCPNGHVCYTK
	P82942.1	RecName: Full=Hemorrhagic metalloproteinase-disintegrin-like kaouthiagin; AltName: Full=Snake venom metalloproteinase; Short=SVMP	SVMP	26	44.5	29.73	0.00495	NGHPCQNNQGYCYNGK<br>IRVYEMINAVNTK
	AAB25732.1	cardiotoxin isoform 1, cytotoxin isoform 1, CTX-1 [*Naja naja* =Formosan cobra, ssp. atra, venom, Peptide, 60 aa]	Cardiotoxin	77	6.7	28.11	0.00006	MFMMSDLTIPVKR<br>GCIDVCPKSNLLVK
	P60308.1	RecName: Full=Cytotoxin SP15c	3FTx	40	6.8	26.8	0.00023	KLVPLFSK<br>ECIDVCPK
	ADF43026.1	metalloproteinase atrase A [*Naja atra*]	SVMP	15	68.2	25.19	0.00599	VYEMVNYLNTK<br>TRVYEMVNYLNTK
	2WQ5	A Chain A, Phospholipase A2, Acidic	PLA2	64	13.3	25.08	0.00003	CCQVHDNCYNEAEK<br>LAAICFAGAPYNDNNYNIDLK
	JAS05143.1	ecto-5'-nucleotidase [*Micrurus tener*]	5'-nucleotidase	14	63	23.37	0.00024	LTILHTNDVHAR<br>VPTYVPLQMEK<br>IINVGSEK
	1CXN	A Chain A, CARDIOTOXIN GAMMA	Cardiotoxin	35	6.8	22.68	0.00085	GCIDVCPK<br>MTMRAAPMVPVKR
	P25517.3	RecName: Full=Cytotoxin 5; AltName: Full=CTX M5; AltName: Full=CTX V	3FTx	28	6.8	22.61	0.00003	YECCDTDR
	3PVM	D Chain D, Cobra Venom Factor	CVF	12	184.4	22.5	0.00227	QNQYVVVQVTGPQVR<br>INYENAILAR<br>YVLPSFEVR
	P20229.1	RecName: Full=Kunitz-type serine protease inhibitor; AltName: Full=Venom trypsin inhibitor	KUN	58	6.4	21.01	0.02281	RPGFCELPAAK<br>FIYGGCGGNANR<br>AHKPAFYYNK
	5GZ4	A Chain A, Snake Venom Phosphodiesterase (pde)	PDE	17	94.6	17.54	0.00163	NAAWWGGQPIWHTASYQGLK<br>NPFYNPSPAK<br>LNLIDQAK
	AAM51550.1	mocarhagin 1 [*Naja mossambica*]	SVMP	8	68.1	12.89	0.00254	VYEMVNALNTMYR<br>SVAVVQDHSK<br>DRPQCILNKPSR
	ETE68810.1	Glutathione peroxidase 3, partial [*Ophiophagus hannah*]	Peroxidase	22	29.6	12.25	0.00134	NSCPPVVETFGDTAK<br>QEPGQNSEILQGIK<br>FLVNPQGKPVMR
	P84805.2	RecName: Full=Cysteine-rich venom protein kaouthin-1; AltName: Full=Cysteine-rich venom protein 25; Short=CRVP-25k; Flags: Precursor	CRISP	39	26.8	11.21	0.00491	QKEIVDLHNSLR<br>MEWYPEAASNAER<br>LGPPCGDCPSACDNGLCTNPCTIYNK
	P01427.1	RecName: Full=Short neurotoxin 1; AltName: Full=Neurotoxin II; Short=NT II; Short=NTII; Short=NTX II; AltName: Full=Neurotoxin alpha	3FTx	61	6.9	10.02	0.01013	VKPGVNLNCCR<br>TCSGETNCYKK<br>LECHNQQSSQPPTTK
	P29180.1	RecName: Full=Weak neurotoxin 6	3FTx	63	7.6	9.8	0.00385	LTCLICPEK<br>VHTCLNGEK<br>EIVQCCSTDK
	AFJ59923.1	OVF precursor protein [*Ophiophagus hannah*]	Complement C3	3	183.8	9.4	0.00023	KLDDKVPDTEIETK<br>HFEVGFIQPGSVK
	JAI08992.1	Metalloproteinase (type III) 1 [*Micrurus fulvius*]	SVMP	6	69	8.19	0.00018	CGDGMVCSNR<br>CPTDSFQR
	AAP20603.1	cysteine-rich venom protein [*Naja atra*]	CRISP	12	26.2	7.92	0.00061	QNACQTEWMK<br>CAASCFCR<br>CSFAHSPPHLR
	P82463.1	RecName: Full=Muscarinic toxin-like protein 2; Short=MTLP-2	3FTx	80	7.3	7.44	0.00341	WHMLVPGR<br>SIFGVTTEDCPDGQNLCFK<br>GCAATCPIAENR
	3HRZ	A Chain A, Cobra Venom Factor	CVF	9	69.5	7.4	0.00038	ALYTLITPAVLR<br>YFTYLILNK
	P82464.1	RecName: Full=Muscarinic toxin-like protein 3; Short=MTLP-3	3FTx	86	7.6	4.55	0.00359	ISLADGNDVR<br>GCTFTCPELRPTGIYVYCCR<br>TICYNHLTR
	P82885.1	RecName: Full=Thaicobrin	SVMP	37	12	3.94	0.00047	FDGSPCVLGSPGFR<br>TVENVGVSQVAPDNPERFDGSPCVLGSPGFR

								EWAVGLAGK
	1MH2	A Chain A, Phospholipase A2	PLA2	27	13.1	3.49	0.00365	DFADYGCYCGR
	AAX86641.1	venom factor [*Austrelaps superbus*]	CVF	2	184.8	2.55	0.00005	KVEGVAFVLFGVK<br>GNANSLNQIK
	ETE56723.1	Endonuclease domain-containing 1 protein [*Ophiophagus hannah*]	Endonuclease	47	18.1	2.53	0.00016	VNRPSHLWSAACCLIDNNHLR<br>LAQLYNVNHVSLFHSDCPR<br>GHLNPNGHQPDYSAK<br>GCQQTFAVVGAVPGDTYIAR
	1FFJ	A Chain A, Nmr Structure Of Cardiotoxin In Dpc-Micelle	Cardiotoxin	62	6.6	90.55	0.08044	MFMVAAPHVPVKR<br>RGCIDVCPK<br>YVCCNTDK
	P25668.1	RecName: Full=Long neurotoxin 1; AltName: Full=Toxin A	3FTx	63	7.8	82.63	0.48047	TWCDGFCSIR<br>VDLGCAATCPTVR<br>DCPNGHVCYTK
	4AEA	A Chain A, Long Neurotoxin 1	3FTx	76	7.8	64.44	0.07668	VDLGCAATCPTVK<br>TGVDIQCCSTDNCNPFPTR
	P25672.1	RecName: Full=Long neurotoxin 4; AltName: Full=Toxin D	3FTx	63	7.9	54.19	0.11952	TWCDGFCR<br>GERVDLGCAATCPTVK
	1KXI	B Chain B, Structure Of Cytotoxin Homolog Precursor	3FTx	53	7	40.3	0.03578	LKCHNTQLPFIYK<br>CHNTQLPFIYK<br>KFPLKFPVK
	CAA45372.1	phospholipase a2 [*Naja naja*]	PLA2	63	13.5	29.14	0.00885	TYSYECSQGTLTCK<br>CCQVHDNCYNEAEK<br>LAAICFAGAPYNDNNYNIDLK
	5H7W	A Chain A, venom 5'-nucleotidase	5'-nucleotidase	20	58.2	24.31	0.00136	IINVGSEK<br>IIALGHSGFMEDCR<br>QVPVVQAYAFGK
	BAA36404.1	phospholipase A2 [*Naja kaouthia*]	PLA2	49	16	24.27	0.08263	NMIQCTVPSR<br>LAAICFAGAPYNNNNYNIDLK
	JAS05143.1	ecto-5'-nucleotidase [*Micrurus tener*]	5'-nucleotidase	16	63	21.29	0.00016	LTILHTNDVHAR<br>VPTYVPLQMEK
	5Z2G	A Chain A, L-amino acid oxidase	LAAO	18	57.9	21.26	0.00221	IHFAGEYTGR<br>SASQLYQESLRK<br>RIYFEPPLPPK
	P60309.1	RecName: Full=Cytotoxin SP15d	3FTx	42	6.6	20.87	0.00204	RGCINVCPK<br>KLVPLFSK
	2WQ5	A Chain A, Phospholipase A2, Acidic	PLA2	52	13.3	20.73	0.00221	GGNNACAASVCDCDR
	P20229.1	RecName: Full=Kunitz-type serine protease inhibitor; AltName: Full=Venom trypsin inhibitor	KUN	58	6.4	19.91	0.01787	RPGFCELPAAK<br>AHKPAFYYNK<br>FIYGGCGGNANR
	CAM34525.2	phospholipase A2, partial [*Naja atra*]	PLA2	44	12.3	19.84	0.00032	MIQCTVPSR
	D3TTC2.1	RecName: Full=Zinc metalloproteinase-disintegrin-like atragin; AltName: Full=Snake venom metalloproteinase; Short=SVMP; Flags: Precursor	SVMP	14	69.1	18.53	0.00508	TKPAYQFSSCSVR<br>RTKPAYQFSSCSVR<br>NSMICNCSISPR
Naja naja (Himachal Pradesh)	ADF43026.1	metalloproteinase atrase A [*Naja atra*]	SVMP	8	68.2	16.48	0.00316	TGCIVPVSPR<br>TRVYEMVNYLNTK<br>CGTLYCTEIK
	3PVM	D Chain D, Cobra Venom Factor	CVF	8	184.4	16.37	0.00153	INYENAILAR<br>LNQDITVTASGDGK<br>IDVPLQIEK<br>ICIGNVCR
	P01427.1	RecName: Full=Short neurotoxin 1; AltName: Full=Neurotoxin II; Short=NT II; Short=NTII; Short=NTX II; AltName: Full=Neurotoxin alpha	3FTx	70	6.9	16.21	0.03688	VKPGVNLNCCR<br>LECHNQQSSQPPTTK<br>TCSGETNCYKK
	AAF00693.1	cobrin precursor [*Naja naja*]	SVMP	11	67.6	15.55	0.00045	TSAAVVQDYSK
	5GZ4	A Chain A, Snake Venom Phosphodiesterase (pde)	PDE	11	94.6	15.17	0.00101	NPFYNPSPAK<br>SMEAIFLAHGPGFK<br>TPLNCPPGSLK
	P29180.1	RecName: Full=Weak neurotoxin 6	3FTx	69	7.6	10.91	0.00709	LTCLICPEKYCNK<br>VHTCLNGEKICFK<br>EIVQCCSTDK
	1XTA	A Chain A, Crystal Structure Of Natrin, A Snake Venom Crisp From Taiwan Cobra (*Naja atra*)	CRISP	27	24.9	9.05	0.00232	SNCPASCFCR<br>QKEIVDLHNSLR<br>NVDFNSESTR
	AFJ59923.1	OVF precursor protein [*Ophiophagus hannah*]	CVF	3	183.8	7.98	0.00039	TDTEEQILVEAHGDNTPK<br>KLDDKVPDTEIETK<br>HFEVGFIQPGSVK
	AAA90960.1	cardiotoxin 1e [*Naja atra*]	Cardiotoxin	22	10.8	7.77	0.00018	MFMMSDLTIPVKR<br>GCIDVCPK
	BAU24669.1	cytotoxin 12, partial [*Naja naja*]	3FTx	32	8	5.67	0.00259	NSLLVKYECCNTDR<br>YECCNTDR
	AAK49439.1	cardiotoxin [*Naja sputatrix*]	3FTx	21	9	5.42	0.02488	MYMVATPK

	P82463.1	RecName: Full=Muscarinic toxin-like protein 2; Short=MTLP-2	3FTx	77	7.3	5.24	0.00288	SIFGVTTEDCPDGQNLCFKR<br>DVIECCSTDK<br>GCAATCPIAENR
	P82942.1	RecName: Full=Hemorrhagic metalloproteinase-disintegrin-like kaouthiagin; AltName: Full=Snake venom metalloproteinase; Short=SVMP	SVMP	17	44.5	4.35	0.00073	IRVYEMINAVNTK<br>CPTLTNQCIALLGPHFTVSPK<br>HDCDLPELCTGQSAECPTDSLQR
	3HRZ	A Chain A, Cobra Venom Factor	CVF	5	69.5	2.42	0.00029	VGLVAVDK<br>YFTYLILNK<br>ALYTLITPAVLR
Naja naja (Irula)	P25668.1	RecName: Full=Long neurotoxin 1; AltName: Full=Toxin A	3FTx	66	7.8	110.8	0.3795	RVDLGCAATCPTVR<br>TWCDGFCSIR
	4AEA	A Chain A, Long Neurotoxin 1	3FTx	76	7.8	86.42	0.0743	TGVDIQCCSTDNCNPFPTR<br>VDLGCAATCPTVK<br>DCPNGHVCYTK
	P25672.1	RecName: Full=Long neurotoxin 4; AltName: Full=Toxin D	3FTx	63	7.9	72.47	0.0634	TWCDGFCR<br>GERVDLGCAATCPTVK
	P86538.2	RecName: Full=Cytotoxin 2a; Short=CTX2a; AltName: Full=Cytotoxin 2; Short=CTX2	3FTx	62	6.7	52	0.1474	NSLLVKYECCNTDR<br>MFMVSDLTIPVK<br>GCIDVCPK
	1KXI	B Chain B, Structure Of Cytotoxin Homolog Precursor	CVF	53	7	51.56	0.0474	YVCCSTDK<br>CHNTQLPFIYK<br>KFPLKFPVK
	3PVM	D Chain D, Cobra Venom Factor	CVF	18	184.4	50.67	0.0056	QLDIFVHDFPR<br>IPIIDGDGK<br>MVAGISHEIICGGVR
	D3TTC2.1	RecName: Full=Zinc metalloproteinase-disintegrin-like atragin; AltName: Full=Snake venom metalloproteinase; Short=SVMP; Flags: Precursor	SVMP	22	69.1	45.07	0.0043	NSMICNCSISPR<br>NGLPCQNNQGYCYNGK<br>RNSMICNCSISPR
	5Z2G	A Chain A, L-amino acid oxidase	LAAO	23	57.9	40.1	0.0031	REIQALCYPSIK<br>SASQLYQESLRK<br>VTLLEASER
	P82942.1	RecName: Full=Hemorrhagic metalloproteinase-disintegrin-like kaouthiagin; AltName: Full=Snake venom metalloproteinase; Short=SVMP	SVMP	34	44.5	37.6	0.0178	RTAPAFQFSSCSIR<br>NGHPCQNNQGYCYNGK<br>IRVYEMINAVNTK
	AAF00693.1	cobrin precursor [*Naja naja*]	SVMP	24	67.6	37.15	0.0196	AAKDDCDLPELCTGQSAECPTDVFQR<br>CPIMTNQCIALR
	AFJ59923.1	OVF precursor protein [*Ophiophagus hannah*]	CVF	9	183.8	34	0.0013	VPVVSEAIHSEGTTLSDGTAK<br>TDTEEQILVEAHGDNTPK<br>VAVIIYLDK<br>KLDDKVPDTEIETK
	CAA45372.1	phospholipase a2 [*Naja naja*]	PLA2	46	13.5	32.9	0.0785	TYSYECSQGTLTCK<br>CCQVHDNCYNEAEK<br>TYSYECSQGTLTCKGDNNACAASVCDCDR
	ADF43026.1	metalloproteinase atrase A [*Naja atra*]	SVMP	13	68.2	30.32	0.0078	IPCAAKDEK<br>VYEMVNYLNTK
	CAM34525.2	phospholipase A2, partial [*Naja atra*]	PLA2	44	12.3	26.45	0.0008	MIQCTVPSR
	ACN50005.1	K-like metalloprotease precursor, partial [*Naja atra*]	SVMP	13	66.2	25.92	0.0005	YYNNDKPAIK<br>VAKDDCDLPELCTGQSAECPTDSLQR<br>DDCDLPELCTGQSAECPTDSLQR<br>ETVLLPR
	AAK49439.1	cardiotoxin [*Naja sputatrix*]	Cardiotoxin	38	9	24.09	0.0705	LVPLFYK<br>MYMVATPK
	PSNJ3K	phospholipase A2 (EC 3.1.1.4) III - monocled cobra	PLA2	42	13.3	21.87	0.0272	NMIQCTVPSR<br>GGSGTPVDDLDR
	5H7W	A Chain A, venom 5'-nucleotidase	5'-Nucleotidase	20	58.2	21.42	0.0019	NVKFPILSANIRPK<br>QVPVVQAYAFGK<br>VPTYVPLEMEK
	2WQ5	A Chain A, Phospholipase A2, Acidic	PLA2	34	13.3	20.42	0.0001	GGNNACAASVCDCDR
	JAS05143.1	ecto-5'-nucleotidase [*Micrurus tener*]	5'-Nucleotidase	16	63	19.35	0.0002	LTILHTNDVHAR<br>VPTYVPLQMEK
	AAB18383.1	cardiotoxin 3a [*Naja atra*]	Cardiotoxin	46	9.1	18.95	0.0075	MFMVATPK<br>YVCCNTDR
	P01447.1	RecName: Full=Cytotoxin 1; AltName: Full=Cobramine-A; AltName: Full=Cytotoxin I; AltName: Full=Cytotoxin XI; AltName: Full=Cytotoxin-like basic protein; Short=CLBP	3FTx	62	6.8	17.76	0.0005	NSLVLKYECCNTDR<br>MYMVSNK
	3HRZ	A Chain A, Cobra Venom Factor	CVF	15	69.5	17.44	0.0008	DTCMGTLVVK<br>ALYTLITPAVLR
	P29180.1	RecName: Full=Weak neurotoxin 6	3FTx	72	7.6	15.52	0.0038	LTCLICPEK<br>VHTCLNGEKICFK<br>GCADTCPVRKPR
	ETE68810.1	Glutathione peroxidase 3, partial [*Ophiophagus hannah*]	Peroxidase	24	29.6	15.39	0.0016	NSCPPVVETFGDTAK<br>QEPGQNSEILQGIK<br>TNVSTVKNDIIR

P01427.1	RecName: Full=Short neurotoxin 1; AltName: Full=Neurotoxin II; Short=NT II; Short=NTII; Short=NTX II; AltName: Full=Neurotoxin alpha	3FTx	61	6.9	14.91	0.0137	VKPGVNLNCCR<br>TCSGETNCYK<br>TCSGETNCYKK
5GZ4	A Chain A, Snake Venom Phosphodiesterase (pde)	PDE	9	94.6	14.78	0.0011	NPFYNPSPAK<br>SMEAIFLAHGPGFK<br>TLGMLMEGLK
P20229.1	RecName: Full=Kunitz-type serine protease inhibitor; AltName: Full=Venom trypsin inhibitor	KUN	58	6.4	13.72	0.0112	RPGFCELPAAK<br>AHKPAFYYNK<br>FIYGGCGGNANR
P84805.2	RecName: Full=Cysteine-rich venom protein kaouthin-1; AltName: Full=Cysteine-rich venom protein 25; Short=CRVP-25k; Flags: Precursor	CRISP	32	26.8	12.99	0.0031	QKEIVDLHNSLR<br>MEWYPEAASNAER<br>WANTCSLNHSPDNLR
P00600.1	RecName: Full=Acidic phospholipase A2 DE-II; Short=svPLA2; AltName: Full=Phosphatidylcholine 2-acylhydrolase	PLA2	18	13.4	12.29	0.0006	ISGCWPYIK
JAI08992.1	Metalloproteinase (type III) 1 [*Micrurus fulvius*]	SVMP	6	69	11.65	0.0002	CGDGMVCSNR<br>CPTDSFQR
AAM51550.1	mocarhagin 1 [*Naja mossambica*]	SVMP	10	68.1	11.58	0.0005	NDCDFPELCTGR<br>VYEMVNALNTMYR<br>SVAVVQDHSK
AAB19290.1	miscellaneous type neurotoxin [*Naja naja* =cobra, ssp. naja, Peptide, 65 aa]	3FTx	35	7.6	9.14	0.0001	RGCAATCPEAKPR<br>EIVQCCSTDK<br>GCAATCPEAKPR
P82463.1	RecName: Full=Muscarinic toxin-like protein 2; Short=MTLP-2	3FTx	46	7.3	8.69	0.0016	WHMLVPGR<br>DVIECCSTDK<br>GCAATCPIAENR
AAP20603.1	cysteine-rich venom protein [*Naja atra*]	CRISP	19	26.2	7.7	0.0003	NMLQMEWNSNAAQNAK<br>QNACQTEWMK<br>CSFAHSPPHLR<br>CAASCFCR
P82464.1	RecName: Full=Muscarinic toxin-like protein 3; Short=MTLP-3	3FTx	60	7.6	4.36	0.0025	ISLADGNDVR<br>GCTFTCPELRPTGIYVYCCR<br>TICYNHLTR
P25668.1	RecName: Full=Long neurotoxin 1; AltName: Full=Toxin A	3FTx	66	7.8	88.63	0.28715	RVDLGCAATCPTVR<br>TWCDGFCSIR<br>GKRVDLGCAATCPTVR
AAB24494.1	Vc-5=cytotoxin [Naja oxiana=snakes, venom, Peptide, 60 aa]	3FTx	62	6.7	83.61	0.03715	MFMVAAPHVPVK<br>KLVPLFSK<br>GCIDVCPK
4AEA	A Chain A, Long Neurotoxin 1	3FTx	76	7.8	75.34	0.11711	TGVDIQCCSTDNCNPFPTR<br>VDLGCAATCPTVK<br>DCPNGHVCYTK
P86538.2	RecName: Full=Cytotoxin 2a; Short=CTX2a; AltName: Full=Cytotoxin 2; Short=CTX2	3FTx	58	6.7	51.64	0.14840	NSLLVKYECCNTDR<br>YECCNTDR
3PVM	D Chain D, Cobra Venom Factor	CVF	16	184.4	47.86	0.00746	QLDIFVHDFPR<br>VYSYYNLDEK<br>WPHEDECQEEEFQK
5H7W	A Chain A, venom 5'-nucleotidase	5'-nucleotidase	27	58.2	40.55	0.00505	NVKFPILSANIRPK<br>QVPVVQAYAFGK<br>SIQEDPAVKAEISR
BAA36404.1	phospholipase A2 [*Naja kaouthia*]	PLA2	55	16	33.73	0.18311	NMIQCTVPSR<br>TYSYECSQGTLTCK<br>CCQVHDNCYNEAEK
1KXI	B Chain B, Structure Of Cytotoxin Homolog Precursor	3FTx	53	7	33.33	0.01401	YVCCSTDKCN<br>CHNTQLPFIYK<br>FPLKFPVK
CAA45372.1	phospholipase a2 [*Naja naja*]	PLA2	71	13.5	30.34	0.00542	GDNNACAASVCDCDR<br>LAAICFAGAPYNDNNYNIDLK
JAS05143.1	ecto-5'-nucleotidase [*Micrurus tener*]	5'-nucleotidase	20	63	29.69	0.00040	VPTYVPLQMEK<br>LTILHTNDVHAR
3K7L	A Chain A, Structures Of Two Elapid Snake Venom Metalloproteases With Distinct Activities Highlight The Disulfide Patterns In The D Domain Of Adamalysin Family Proteins	SVMP	33	47.7	29.56	0.00497	NSMICNCSISPR<br>RTKPAYQFSSCSVR<br>DPNYGMVEPGTK
CAM34525.2	phospholipase A2, partial [*Naja atra*]	PLA2	52	12.3	27.54	0.00224	MIQCTVPSR
AFJ59923.1	OVF precursor protein [*Ophiophagus hannah*]	CVF	6	183.8	27.04	0.00066	TDTEEQILVEAHGDNTPK<br>YEVDNNMAQK
AAF00693.1	cobrin precursor [*Naja naja*]	SVMP	22	67.6	23.15	0.01753	TSAAVVQDYSK<br>DPSYGMVEPGTK<br>TKPAYQFSSCSVR
2WQ5	A Chain A, Phospholipase A2, Acidic	PLA2	52	13.3	23.12	0.01121	GGSGTPVDDLDR
5GZ4	A Chain A, Snake Venom Phosphodiesterase (pde)	PDE	13	94.6	22.23	0.00206	NPFYNPSPAK<br>RPDFSTLYIEEPDTTGHK<br>SMEAIFLAHGPGFK

Naja naja (West Bengal): Sample A	ADF43026.1	metalloproteinase atrase A [*Naja atra*]	SVMP	12	68.2	19.36	0.01234	NGHPCQNNQGYCYNGK<br>VYEMVNYLNTK<br>ERPQCILNKPSR
	P60308.1	RecName: Full=Cytotoxin SP15c	SVMP	57	6.8	18.97	0.01562	MFMVATPK<br>YVCCNTDK
	AAK49439.1	cardiotoxin [*Naja sputatrix*]	Cardiotoxin	30	9	18.87	0.05776	MYMVATPK<br>YVCCNTDR
	P82942.1	RecName: Full=Hemorrhagic metalloproteinase-disintegrin-like kaouthiagin; AltName: Full=Snake venom metalloproteinase; Short=SVMP	SVMP	16	44.5	18.27	0.01571	VYEMINAVNTK<br>DYQEYLLR<br>IRVYEMINAVNTK
	5Z2G	A Chain A, L-amino acid oxidase	LAAO	19	57.9	18.27	0.00152	SASQLYQESLR<br>VWEVKKDPSLLK<br>YPVKPSEEGK
	3HRZ	A Chain A, Cobra Venom Factor	CVF	15	69.5	16.93	0.00035	YFTYLILNK<br>VGLVAVDK
	AAX86641.1	venom factor [*Austrelaps superbus*]	CVF	4	184.8	16.17	0.00037	YFTYLILTK<br>DTCMGTLVVK
	P20229.1	RecName: Full=Kunitz-type serine protease inhibitor; AltName: Full=Venom trypsin inhibitor	KUN	58	6.4	16.03	0.01201	RPGFCELPAAK<br>FIYGGCGGNANR<br>AHKPAFYYNK
	P01447.1	RecName: Full=Cytotoxin 1; AltName: Full=Cobramine-A; AltName: Full=Cytotoxin I; AltName: Full=Cytotoxin XI; AltName: Full=Cytotoxin-like basic protein; Short=CLBP	3FTx	58	6.8	14.51	0.00019	NSLVLKYECCNTDR<br>MYMVSNKTVPVKR
	AAP20603.1	cysteine-rich venom protein [*Naja atra*]	CRISP	19	26.2	13	0.00180	NMLQMEWNSNAAQNAK<br>QNACQTEWMK<br>CSFAHSPPHLR
	P82463.1	RecName: Full=Muscarinic toxin-like protein 2; Short=MTLP-2	3FTx	77	7.3	11.37	0.00411	SIFGVTTEDCPDGQNLCFKR<br>WHMLVPGR<br>DVIECCSTDK
	P01460.1	RecName: Full=Cytotoxin 8; AltName: Full=Toxin CM-7	3FTx	30	6.8	11.08	0.00121	YVCCNTNK<br>YVCCNTNKCN
	AAM51550.1	mocarhagin 1 [*Naja mossambica*]	SVMP	6	68.1	10.45	0.00075	NDCDFPELCTGR<br>SVAVVQDHSK<br>AAKNDCDFPELCTGR
	P84805.2	RecName: Full=Cysteine-rich venom protein kaouthin-1; AltName: Full=Cysteine-rich venom protein 25; Short=CRVP-25k; Flags: Precursor	CRISP	32	26.8	9.55	0.00455	QKEIVDLHNSLR<br>SNCPASCFCR<br>MEWYPEAASNAER
	AAB19290.1	miscellaneous type neurotoxin [*Naja naja* =cobra, ssp. naja, Peptide, 65 aa]	3FTx	35	7.6	8.42	0.00022	RGCAATCPEAKPR<br>EIVQCCSTDK<br>GCAATCPEAKPR
	JAI08992.1	Metalloproteinase (type III) 1 [*Micrurus fulvius*]	SVMP	6	69	8.02	0.00014	CGDGMVCSNR<br>CPTDSFQR
	P01427.1	RecName: Full=Short neurotoxin 1; AltName: Full=Neurotoxin II; Short=NT II; Short=NTII; Short=NTX II; AltName: Full=Neurotoxin alpha	3FTx	61	6.9	7.14	0.02556	TCSGETNCYKK<br>VKPGVNLNCCR<br>LECHNQQSSQPPTTK
	P82464.1	RecName: Full=Muscarinic toxin-like protein 3; Short=MTLP-3	3FTx	29	7.6	4.71	0.00182	TICYNHLTR<br>ISLADGNDVR
	A8QL48.1	RecName: Full=Zinc metalloproteinase-disintegrin-like BfMP; AltName: Full=Snake venom metalloproteinase; Short=SVMP; Flags: Precursor	SVMP	5	68.2	3.97	0.00002	NGLPCQNNQGYCYNGK<br>DSCFTLNQR<br>FSSCSVR
	P25668.1	RecName: Full=Long neurotoxin 1; AltName: Full=Toxin A	3FTx	69	7.8	138.65	0.50452	RVDLGCAATCPTVR<br>IRCFITPDITSK<br>VDLGCAATCPTVR
	P25669.1	RecName: Full=Long neurotoxin 2; AltName: Full=Toxin B	3FTx	69	7.8	137.81	0.04599	TWCDGFCSSR
	4AEA	A Chain A, Long Neurotoxin 1	3FTx	79	7.8	95.46	0.16695	TGVDIQCCSTDNCNPFPTR<br>DCPNGHVCYTK<br>CFITPDITSK
	P25672.1	RecName: Full=Long neurotoxin 4; AltName: Full=Toxin D	3FTx	55	7.9	81.19	0.00361	VDLGCAATCPTVK<br>GERVDLGCAATCPTVK
	3PVM	D Chain D, Cobra Venom Factor	CVF	16	184.4	49.98	0.00339	QLDIFVHDFPR<br>QNQYVVVQVTGPQVR<br>LNQDITVTASGDGK
	5Z2G	A Chain A, L-amino acid oxidase	LAAO	33	57.9	48.19	0.00225	REIQALCYPSIK<br>SASQLYQESLR<br>VTYQTPAK
	D3TTC2.1	RecName: Full=Zinc metalloproteinase-disintegrin-like atragin; AltName: Full=Snake venom metalloproteinase; Short=SVMP; Flags: Precursor	SVMP	18	69.1	36.22	0.00353	NSMICNCSISPR<br>RTKPAYQFSSCSVR<br>NGLPCQNNQGYCYNGK
	1KXI	B Chain B, Structure Of Cytotoxin Homolog Precursor	3FTx	53	7	35.9	0.01340	YVCCSTDK<br>CHNTQLPFIYK<br>FPLKFPVK

	CAA45372.1	phospholipase a2 [*Naja naja*]	PLA2	53	13.5	33.49	0.05999	TYSYECSQGTLTCK<br>GGSGTPVDDLDR<br>GDNNACAASVCDCDR
	AFJ59923.1	OVF precursor protein [*Ophiophagus hannah*]	CVF	7	183.8	33.18	0.00137	VSHSEDECLQFK<br>TDTEEQILVEAHGDNTPK<br>KLDDKVPDTEIETK<br>ACASNVDYVYK
	AAF00693.1	cobrin precursor [*Naja naja*]	SVMP	18	67.6	32.88	0.01373	TSAAVVQDYSK<br>DPSYGMVEPGTK<br>ATLDLFGEWR
	ADF43026.1	metalloproteinase atrase A [*Naja atra*]	SVMP	18	68.2	31.96	0.02248	AAKDDCDLPEFCTGQSAECPTDSLQR<br>IPCAAKDEK<br>LQPHAQCDSEECCEK
	P82942.1	RecName: Full=Hemorrhagic metalloproteinase-disintegrin-like kaouthiagin; AltName: Full=Snake venom metalloproteinase; Short=SVMP	SVMP	24	44.5	30.46	0.00452	QTVLLPR<br>IRVYEMINAVNTK<br>CGDGMVCSK
	ACN50005.1	K-like metalloprotease precursor, partial [*Naja atra*]	SVMP	13	66.2	23.21	0.00079	RNDNAQLLTGIDFNGNTVGR<br>NDNAQLLTGIDFNGNTVGR
	P01427.1	RecName: Full=Short neurotoxin 1; AltName: Full=Neurotoxin II; Short=NT II; Short=NTII; Short=NTX II; AltName: Full=Neurotoxin alpha	3FTx	61	6.9	22.6	0.04860	VKPGVNLNCCR<br>TCSGETNCYK<br>LECHNQQSSQPPTTK
	2WQ5	A Chain A, Phospholipase A2, Acidic	PLA2	34	13.3	22.52	0.00026	CCQVHDNCYNEAEK<br>GGNNACAASVCDCDR
	P62394.1	RecName: Full=Cytotoxin 11; AltName: Full=Toxin CM-12	3FTx	37	6.8	20.72	0.00008	VVCCSTDKCN<br>LKCHNTQLPFIYK
Naja naja (West Bengal): Sample B	JAS05143.1	ecto-5'-nucleotidase [*Micrurus tener*]	5'-nucleotidase	18	63	20.71	0.00072	NVKFPILSANIRPK<br>QVPVVQAYAFGK<br>SIQEDPAVK
	AAK49439.1	cardiotoxin [*Naja sputatrix*]	Cardiotoxin	57	9	19.28	0.01902	RGCIDVCPK<br>MYMVATPK<br>YVCCNTDR
	AAB19290.1	miscellaneous type neurotoxin [*Naja naja* =cobra, ssp. naja, Peptide, 65 aa]	3FTx	35	7.6	17.35	0.00172	RGCAATCPEAKPR<br>EIVQCCSTDK<br>GCAATCPEAKPR
	P82463.1	RecName: Full=Muscarinic toxin-like protein 2; Short=MTLP-2	3FTx	85	7.3	16.3	0.00742	SIFGVTTEDCPDGQNLCFK<br>WHMLVPGR<br>DVIECCSTDK
	JAI08992.1	Metalloproteinase (type III) 1 [*Micrurus fulvius*]	SVMP	6	69	16.2	0.00009	NGHPCQNNQGYCYNGK<br>CPTDSFQR<br>CGDGMVCSNR
	3HRZ	A Chain A, Cobra Venom Factor	CVF	13	69.5	15.86	0.00016	ALYTLITPAVLR<br>YFTYLILNK
	BAU24669.1	cytotoxin 12, partial [*Naja naja*]	3FTx	57	8	15.62	0.01151	NSLLVKYECCNTDR<br>YECCNTDR
	5GZ4	A Chain A, Snake Venom Phosphodiesterase (pde)	PDE	9	94.6	15.22	0.00075	NPFYNPSPAK<br>LNLIDQAK<br>SMEAIFLAHGPGFK
	Q01833.1	RecName: Full=Complement C3	CVF	4	184.8	15.18	0.00018	GICVAEPYEITVMK<br>CQEALNLK
	1XTA	A Chain A, Crystal Structure Of Natrin, A Snake Venom Crisp From Taiwan Cobra (*Naja atra*)	CRISP	26	24.9	11.52	0.00202	QKEIVDLHNSLR<br>RVSPTASNMLK<br>MEWYPEAASNAER
	P20229.1	RecName: Full=Kunitz-type serine protease inhibitor; AltName: Full=Venom trypsin inhibitor	KUN	70	6.4	10.58	0.00426	RPGFCELPAAK<br>FIYGGCGGNANR<br>AHKPAFYYNK
	AAA90960.1	cardiotoxin 1e [*Naja atra*]	Cardiotoxin	47	10.8	9.59	0.00975	MFMMSDLTIPVKR<br>TCPAGKNLCYK
	A8QL48.1	RecName: Full=Zinc metalloproteinase-disintegrin-like BfMP; AltName: Full=Snake venom metalloproteinase; Short=SVMP; Flags: Precursor	SVMP	5	68.2	7.9	0.00011	FSSCSVR<br>DSCFTLNQR
	AAB24494.1	Vc-5=cytotoxin [Naja oxiana=snakes, venom, Peptide, 60 aa]	3FTx	67	6.7	7.63	0.00001	MFMVAAPHVPVKR
	LAB19280.1	hypothetical protein, partial [*Micrurus spixii*]		7	61.6	7.16	0.00061	ISNIPLEADYSK<br>TLEKDSVGEPK<br>EITTGITTSKPIPNGR
	AAM51550.1	mocarhagin 1 [*Naja mossambica*]	SVMP	4	68.1	6.66	0.00041	NDCDFPELCTGR<br>SVAVVQDHSK<br>AAKNDCDFPELCTGR
	1CDT	A Chain A, CARDIOTOXIN VII4	Cardiotoxin	37	6.7	5.58	0.04425	RGCINVCPK<br>LIPIAYKTCPEGK<br>GCINVCPK
	P82464.1	RecName: Full=Muscarinic toxin-like protein 3; Short=MTLP-3	3FTx	60	7.6	4.43	0.00154	ISLADGNDVR<br>TICYNHLTR

								GCTFTCPELRPTGIYVYCCR
Naja naja (West Bengal): Sample C	4AEA	A Chain A, Long Neurotoxin 1	3FTx	76	7.8	65.01	0.34504	VDLGCAATCPTVK<br>TGVDIQCCSTDNCNPFPTR<br>DCPNGHVCYTK
	P25668.1	RecName: Full=Long neurotoxin 1; AltName: Full=Toxin A	3FTx	66	7.8	64.93	0.49607	RVDLGCAATCPTVR<br>CFITPDITSKDCPNGHVCYTK<br>GKRVDLGCAATCPTVR
	P25669.1	RecName: Full=Long neurotoxin 2; AltName: Full=Toxin B	3FTx	66	7.8	63.13	0.05929	TWCDGFCSSR<br>CFITPDITSK
	P25672.1	RecName: Full=Long neurotoxin 4; AltName: Full=Toxin D	3FTx	52	7.9	41.14	0.00380	GERVDLGCAATCPTVK
	3PVM	D Chain D, Cobra Venom Factor	CVF	19	184.4	38.11	0.00724	SDFGCTAGSGQNNLGVFEDAGLALT<br>RDGQNLVTMNLHITPDLIPSFR<br>QNQYVVVQVTGPQVR
	1FFJ	A Chain A, Nmr Structure Of Cardiotoxin In Dpc-Micelle	Cardiotoxin	52	6.6	33.42	0.02929	MFMVAAPHVPVKR<br>YVCCNTDKCN<br>KLVPLFSK
	5Z2G	A Chain A, L-amino acid oxidase	LAAO	17	57.9	25.26	0.00176	SASQLYQESLR<br>TCADIVINDLSLIHDLPKR<br>VTLLEASER
	3HRZ	A Chain A, Cobra Venom Factor	CVF	23	69.5	18.16	0.00063	QLDIFVHDFPR<br>LILNIPLNAQSLPITVR<br>ALYTLITPAVLR
	CAA45372.1	phospholipase a2 [*Naja naja*]	PLA2	71	13.5	18.11	0.02121	ISGCWPYFK<br>TYSYECSQGTLTCK<br>CCQVHDNCYNEAEKISGCWPYFK
	ABN72543.1	complement-depleting factor [*Ophiophagus hannah*]	CVF	7	184.2	17.33	0.00102	VAVIIYLDK<br>GIYTPGSPVLYR<br>HFEVGFIQPGSVK
	AAF00693.1	cobrin precursor [*Naja naja*]	SVMP	20	67.6	16.98	0.00021	AAKDDCDLPELCTGQSAECPTDVFQR<br>LQHEAQCDSEECCEK<br>TSAAVVQDYSK
	D3TTC2.1	RecName: Full=Zinc metalloproteinase-disintegrin-like atragin; AltName: Full=Snake venom metalloproteinase; Short=SVMP; Flags: Precursor	SVMP	22	69.1	16.58	0.00611	NGLPCQNNQGYCYNGK<br>DPNYGMVEPGTK
	1KXI	B Chain B, Structure Of Cytotoxin Homolog Precursor	3FTx	34	7	15.97	0.00600	CHNTQLPFIYK<br>LKCHNTQLPFIYK<br>FPLKFPVK
	BAA36404.1	phospholipase A2 [*Naja kaouthia*]	PLA2	48	16	15.51	0.01592	LAAICFAGAPYNNNNYNIDLK
	AAX86641.1	venom factor [*Austrelaps superbus*]	CVF	2	184.8	8.45	0.00004	CQEALNLK<br>KVEGVAFVLFGVK
	JAS05036.1	Ecto-5'-nucleotidase 1c [*Micrurus fulvius*]	5'-nucleotidase	13	62.9	7.64	0.00059	NVKFPILSANIRPK<br>YDAMALGNHEFDNGLNGLLDPLLK<br>FHECNLGNLICDAVVYNNLR
	5GZ4	A Chain A, Snake Venom Phosphodiesterase (pde)	PDE	8	94.6	5.54	0.00049	SMEAIFLAHGPGFK<br>NPFYNPSPAK<br>CSSITDLEAVNQR
	P01400.1	RecName: Full=Weak toxin S4C11	3FTx	35	7.4	5.45	0.00148	LTCLICPEKYCNK<br>LTCLICPEK<br>EIVECCSTDK
	P82463.1	RecName: Full=Muscarinic toxin-like protein 2; Short=MTLP-2	3FTx	80	7.3	2.37	0.00320	SIFGVTTEDCPDGQNLCFKR<br>WHMIVPGR<br>EKSIFGVTTEDCPDGQNLCFK
	P84805.2	RecName: Full=Cysteine-rich venom protein kaouthin-1; AltName: Full=Cysteine-rich venom protein 25; Short=CRVP-25k; Flags: Precursor	CRISP	21	26.8	2.16	0.00064	SNCPASCFCR<br>MEWYPEAASNAER<br>LGPPCGDCPSACDNGLCTNPCTIYNK
	AAB25732.1	cardiotoxin isoform 1, cytotoxin isoform 1, CTX-1 [*Naja naja* =Formosan cobra, ssp. atra, venom, Peptide, 60 aa]	3FTx	58	6.7	122.23	0.59137	RGCIDVCPK<br>MFMMSDLTIPVKR<br>YVCCNTDR
	AAB24494.1	Vc-5=cytotoxin [Naja oxiana=snakes, venom, Peptide, 60 aa]	3FTx	62	6.7	91.72	0.08645	GCIDVCPK<br>KLVPLFSK
	5Z2G	A Chain A, L-amino acid oxidase	LAAO	26	57.9	27.13	0.00497	REIQALCYPSIKK<br>RIYFEPPLPPK<br>SASQLYQESLR
	ADF43026.1	metalloproteinase atrase A [*Naja atra*]	SVMP	12	68.2	26.93	0.02521	VYEMVNYLNTK<br>TRVYEMVNYLNTK<br>TGCIVPVSPR
	P83345.1	RecName: Full=Cytotoxin sagitoxin; AltName: Full=Cardiotoxin sagitoxin	3FTx	45	6.8	22.68	0.00078	CNKLVPLAYK<br>YECCNTDR
	JAS05143.1	ecto-5'-nucleotidase [*Micrurus tener*]	5'-nucleotidase	18	63	22.65	0.00316	NVKFPILSANIRPK<br>QVPVVQAYAFGK<br>VPTYVPLQMEK
	P01382.1	RecName: Full=Alpha-elapitoxin-Nno2a; Short=Alpha-EPTX-Nno2a; AltName: Full=Long neurotoxin 1; AltName: Full=Neurotoxin I; Short=NT I; Short=Ntx-1; Short=Toxin I; AltName: Full=NnoI	3FTx	78	8	22.17	0.10048	VIELGCAATCPTVESYQDIK<br>TWCDAWCGSR<br>TPIPITSETCAPGQNLCYTK

	P82942.1	RecName: Full=Hemorrhagic metalloproteinase-disintegrin-like kaouthiagin; AltName: Full=Snake venom metalloproteinase; Short=SVMP	SVMP	16	44.5	21.09	0.02946	RTAPAFQFSSCSIR<br>VYEMINAVNTK<br>TAPAFQFSSCSIR
	5H7W	A Chain A, venom 5'-nucleotidase	5'-nucleotidase	20	58.2	20.85	0.00115	VPTYVPLEMEK<br>HADKLTTLGVNK
	AAF00693.1	cobrin precursor [*Naja naja*]	SVMP	17	67.6	20.37	0.00596	AAKDDCDLPELCTGQSAECPTDVFQR<br>RTKPAYQFSSCSVR<br>CGDGMVCSNR
	O73859.1	RecName: Full=Cytotoxin 7; AltName: Full=Cardiotoxin-7; Short=CTX-7; Short=Ctx7; Flags: Precursor	Cardiotoxin	58	7.1	20.19	0.00031	MFMMSNKTVPVKR<br>LVPLFYK
	1XTA	A Chain A, Crystal Structure Of Natrin, A Snake Venom Crisp From Taiwan Cobra (*Naja atra*)	CRISP	48	24.9	19.59	0.01415	NVDFNSESTR<br>QKEIVDLHNSLR<br>QSSCQDDWIK
Naja oxiana (Himachal Pradesh)	P01401.1	RecName: Full=Weak toxin CM-11		60	7.5	19.32	0.00014	NGENQCFKR<br>GCAATCPEAKPR<br>LTCLICPEKYCNKVHTCR
	1CXN	A Chain A, CARDIOTOXIN GAMMA	Cardiotoxin	35	6.8	18.45	0.00080	MTMRAAPMVPVKR
	5GZ4	A Chain A, Snake Venom Phosphodiesterase (pde)	PDE	16	94.6	18.23	0.00245	QPLSETLR<br>RPDFSTLYIEEPDTTGHK<br>NPFYNPSPAK
	3K7L	A Chain A, Structures Of Two Elapid Snake Venom Metalloproteases With Distinct Activities Highlight The Disulfide Patterns In The D Domain Of Adamalysin Family Proteins	SVMP	28	47.7	16.24	0.02407	NGLPCQNNQGYCYNGK<br>TNTPEQDRYLQAK
	AAB19290.1	miscellaneous type neurotoxin [*Naja naja* =cobra, ssp. naja, Peptide, 65 aa]	3FTx	40	7.6	15.16	0.00156	RGCAATCPEAKPR<br>EIVQCCSTDK
	P29180.1	RecName: Full=Weak neurotoxin 6	3FTx	60	7.6	11.99	0.01000	VHTCLNGEK<br>LTCLICPEK
	ETE68810.1	Glutathione peroxidase 3, partial [*Ophiophagus hannah*]	Peroxidase	19	29.6	11.49	0.00314	QEPGQNSEILQGIK<br>TNVSTVKNDIIR<br>LVILGFPCNQFGK
	AAM51550.1	mocarhagin 1 [*Naja mossambica*]	SVMP	6	68.1	11.1	0.00557	NDCDFPELCTGR<br>SVAVVQDHSK<br>AAKNDCDFPELCTGR
	AAF82187.1	neutral phospholipase A2 [*Naja sputatrix*]	PLA2	27	16.2	10.1	0.00372	TYSYECSQGTLTCK<br>CCQIHDNCYNEAEK<br>GGSGTPVDDLDR
	3PVM	D Chain D, Cobra Venom Factor	CVF	3	184.4	9.97	0.00045	QLDIFVHDFPR<br>VNDDYLIWGSR<br>VAVIIYLNK
	P82463.1	RecName: Full=Muscarinic toxin-like protein 2; Short=MTLP-2	3FTx	80	7.3	9.92	0.01113	WHMLVPGR<br>SIFGVTTEDCPDGQNLCFK<br>GCAATCPIAENR
	P01427.1	RecName: Full=Short neurotoxin 1; AltName: Full=Neurotoxin II; Short=NT II; Short=NTII; Short=NTX II; AltName: Full=Neurotoxin alpha	3FTx	80	6.9	9.13	0.05057	VKPGVNLNCCR<br>TCSGETNCYK<br>LECHNQQSSQPPTTK
	CAA45372.1	phospholipase a2 [*Naja naja*]	PLA2	42	13.5	8.56	0.02227	GDNNACAASVCDCDR<br>ISGCWPYFK
	P25668.1	RecName: Full=Long neurotoxin 1; AltName: Full=Toxin A	3FTx	34	7.8	4.91	0.00051	RVDLGCAATCPTVR<br>CFITPDITSK<br>VDLGCAATCPTVR
	3HRZ	A Chain A, Cobra Venom Factor	CVF	5	69.5	4.11	0.00018	ALYTLITPAVLR<br>VGLVAVDK
