# Supplementary material for: Multilevel Comparison of Indian Naja Venoms and Their Cross-Reactivity with Indian Polyvalent Antivenoms
Source: Toxins (Basel). 2023 Apr 1;15(4):258. doi: 10.3390/toxins15040258 (PMC10142961; doi:10.3390/toxins15040258)
Supplement: Supplementary file 1 [file toxins-15-00258-s001.zip › Supplementary table S2- anitivenomics data-v2.pdf]

Supplementary Table S2: Assignment of peptide fragments belonging to various snake venom protein families obtained by mass spectrometry in each of the non-retained peaks from the antivenomics experiment. Protein were identified by comparing fragment masses against Elapidae database in NCBI. Only proteins with a SEQUEST score of 1.0 or higher are listed.

Sample ID	Fraction	Accession No.	Protein name	Protein family	MW [kDa]	Coverage (%)	Score Sequest	MS derived sequences
Naja kaouthia (Arunachal Pradesh)	F1	1V6P	B Chain B, Cobrotoxin	3FTx	7	63	33.01	NGIEINCCTTDR<br>LECHNQQSSQTPTTTGCSGGETNCYK
		P60772	Neurotoxin 5	3FTx	6.8	54	20	TCSGETNCYK<br>LNCCTTDR
		P14613	Short neurotoxin 1	3FTx	7	63	6.08	NGIEINCCTTDR<br>LECHNQQSIQTPTTTGCSGGETNCYKK
		P59275	Cobrotoxin-b	3FTx	6.9	59	5.64	TCSGETNCYK<br>VKPGVNLNCCR<br>LECHNQQSSQTPTTK
		BAU24666	Cytotoxin 11	3FTx	8	29	4.38	MFMVSDLTIPVKR<br>MFMVSDLTIPVK<br>YVCCNTDR
		AHZ08817	Short neurotoxin	3FTx	9	10	1.86	GCGCPTVK
	F2	BAU24674	cytotoxin 15, partial	3FTx	8	32	7.35	MYMVSNK<br>GCIDVCPK<br>YVCCNTDR
		AAY89680	L-amino acid oxidase precursor	LAAO	59	3	4.1	VTLLEASER<br>STTDLPSR
		AAB19290	miscellaneous type neurotoxin	3FTx	7.6	31	2.76	DGEKICFK<br>GCAATCPEAKPR
	F3	AAB01542	cardiotoxin VIII, partial	3FTx	8.9	22	13.08	YVCCNTDR<br>MFMVATPK<br>YVCCNTDRCN
		ADN67577	three-finger toxin precursor	3FTx	9.9	10	1.72	VHTCLNGEK
		P25679	RecName: Full=Weak toxin CM-9a	3FTx	7.4	20	1.6	LTCLNCPEMFCGK
	F4	AAB25732	cardiotoxin isoform 1	3FTx	6.7	58	33.61	MFMMSDLTIPVKR<br>RGCIDVCPK<br>YVCCNTDR<br>GCIDVCPK<br>GCIDVCPKSNLLVK
		BAU24670	cytotoxin 13, partial	3FTx	7.9	67	30.26	MFMVSNK<br>NLCYKMFMVSNK<br>RGCIDVCPK<br>YVCCNTDR<br>GCIDVCPK<br>GCIDVCPKNSLLVK<br>LIPLAYKTCPAGK
		1CDT	A Chain A, CARDIOTOXIN VII4	3FTx	6.7	37	13.98	RGCINVCPK<br>LIPIAYKTCPEGK<br>GCINVCPK
		P60308	Cytotoxin SP15c	3FTx	6.8	27	4.08	MFMVATPK<br>ECIDVCPK
		A59218	Nerve growth factor beta chain precursor - monocled cobra	NGF	27.6	8	1.62	QYFFETK<br>ALTMEGNQASWR
	F5	P86541	Cytotoxin 10	3FTx	6.8	83	57.33	SSLLVKYVCCNTDR<br>MYMVATPK<br>YVCCNTDRCN<br>YVCCNTDR<br>GCIDVCPK<br>CNKLVPLFYK<br>LVPLFYKTCPAGK<br>LVPLFYK<br>LKCNKLVPLFYK
		P07525	cytotoxin D1	3FTx	6.8	82	44.36	SSLLVKYVCCNTDR<br>MFMVSNK<br>YVCCNTDRCN<br>YVCCNTDR<br>GCIDVCPK<br>CNKLVPLFYK<br>LVPLFYKTCPAGK<br>LVPLFYK<br>LKCNKLVPLFYK
		P86538	Cytotoxin 2a	3FTx	6.7	48	20.34	MFMVSDLTIPVKR<br>YECCNTDR<br>GCIDVCPK
		BAA36404	phospholipase A2	PLA_2	16	51	8.47	NMIQCTVPSR<br>TYSYECSQGTLTCK<br>CCQVHDNCYNEAEK<br>ISGCWPYFK<br>GGSGTPVDDLDR<br>GGNNACAAAVCDCDR
		AAB18387	cardiotoxin 7	3FTx	9.3	13	2.31	CHNTQLPFIYK
		Q9DEQ3	Neurotoxin homolog NL1	3FTx	10	16	2.1	GCTFTCPELRPTGK

Naja kaouthia (Mizoram)	F1	P59276	Cobrotoxin-c	3FTx	6.9	85	18.33	VKPGVNLNCCRTDR<br>VKPGVNLNCCR<br>TCSGETNCYKK<br>LECHNQQSSQAPTTK<br>KWWSDHRGTIIER<br>WWSDHRGTIIER
		AHZ08817	short neurotoxin	3FTx	9	10	16.12	GCGCPTVK
		1V6P	B Chain B, Cobrotoxin	3FTx	7	61	7.33	LECHNQQSSQTPTTTGCSGGETNCYK<br>NGIEINCCTTDR
		P14613	Toxin C-6	3FTx	7	61	4.5	NGIEINCCTTDR<br>LECHNQQSIQTPTTTGCSGGETNCYK
		P59275	Cobrotoxin-b	3FTx	6.9	80	18.26	VKPGVNLNCCR<br>TCSGETNCYKK<br>LECHNQQSSQTPTTK<br>WWSDHRGTIIER
		1CDT	A Chain A, CARDIOTOXIN VII4	3FTx	6.7	13	1.74	GCINVCPK
	F2	2OSH	A Chain A, Phospholipase A2	PLA_2	13.1	67	30.02	NLYQFK<br>NMIQCTVPSR<br>TYSYECSQGTLTCK<br>CCQVHDNCYNEAEK<br>CCQVHDNCYNEAEKISGCWPYFK<br>GGNNACAAAVCDCDR<br>GGSGTPVDDLDR<br>ISGCWPYFK
		Q9I900	Acidic phospholipase A2 D	PLA_2	16.1	45	12.26	NMIQCTVPNR<br>TYSYECSQGTLTCK<br>CCQVHDNCYGEAEK<br>GGNDACAAAVCDCDR<br>GGSGTPVDDLDR
		P60044	Acidic phospholipase A2 2	PLA_2	14.1	29	10.71	NMISCTVPSR<br>CCQVHDNCYNEAEK<br>GGSGTPVDDLDR
		BAU24666	cytotoxin 11	3FTx	8	43	9.74	MFMVSDLTIPVKR<br>GCIDVCPK<br>YVCCNTDR<br>YVCCNTDRCN
		1OWS	A Chain A, Phospholipase A2	PLA_2	13.1	20	3.91	NMIECTVPSR<br>TYTYQCSGGTLTCK
		AAR10440	cardiotoxin-like protein	3FTx	9.3	21	2.08	GCIDICPK<br>KLPSKYDVIR
		1MH2	A Chain A, Phospholipase A2	PLA_2	13.1	9	1.86	DFADYGCYCGR
	F3	1H0J	A Chain A, Structural Basis Of The Membrane-Induced Cardiotoxin A3 Oligomerization	3FTx	6.7	100	75.87	NLCYKMFMVATPK<br>YVCCNTDR<br>VPVKRGCIDVCPK<br>TCPAGKNLCYK<br>MFMVATPKVPVKR<br>GCIDVCPKSSLLVK<br>GCIDVCPK<br>CNKLVPLFYK<br>LKCNKLVPLFYK<br>MFMVATPKVPVK<br>MFMVATPK<br>LVPLFYKTCPAGK<br>LVPLFYK<br>YVCCNTDRCN
		P01440	Cytotoxin 2	3FTx	6.8	90	69.5	MYMVATPKVPVKR<br>MYMVATPKVPVK<br>MYMVATPK<br>NLCYKMYMVATPK<br>YVCCNTDR<br>VPVKRGCIDVCPK<br>TCPAGKNLCYK<br>GCIDVCPK<br>CNKLVPLFYK<br>LKCNKLVPLFYK<br>LVPLFYKTCPAGK<br>LVPLFYK<br>YVCCNTDRCN
		P01446	Cytotoxin 3	3FTx	6.7	87	15.63	NLCYKMFMVSNK<br>MFMVSNKTVPVKR<br>MFMVSNK<br>YVCCNTDR<br>TCPAGKNLCYK<br>CNKLIPLAYK<br>GCIDACPK<br>YVCCNTDRCN
		AAA90960	cardiotoxin 1e	3FTx	10.8	43	8.78	YVCCNTDR<br>TCPAGKNLCYK<br>GCIDVCPK<br>MFMMSDLTIPVKR<br>YVCCNTDRCN
		P60309	Cytotoxin SP15d	3FTx	6.6	32	6.19	VPVKRGCINVCPK<br>GCINVCPKSSLLVK<br>GCINVCPK

		BAU24666	cytotoxin 11, partial	3FTx	8	43	6.19	YVCCNTDR<br>MFMVSDLTIPVKR<br>GCIDVCPK<br>YVCCNTDRCN
		PSNJ2K	phospholipase A2	PLA_2	13.4	34	6.14	NGNNACAAAVCDCDR<br>TYSYECSQGTLTCK<br>GGSGTPVDDLDR
		BAA36404	phospholipase A2	PLA_2	16	40	4.36	TYSYECSQGTLTCK<br>NMIQCTVPSR<br>GGSGTPVDDLDR<br>CCQVHDNCYNEAEK<br>ISGCWPYFK
		AAA66029	phospholipase A2	PLA_2	16.1	27	4.36	TYSYECSQGTLTCK<br>GGSGTPVDDLDR<br>CCQVHDNCYGEAEK
		Q9W717	Neurotoxin-like protein NTL2	3FTx	9.7	12	2	GITRLPWVIR
	F4	P60307	Cytotoxin SP15a	3FTx	6.7	48	50.68	MFMVATPKVPVK<br>MFMVATPKVPVKR<br>MFMVATPK<br>GCIDVCPK<br>KLVPLFSK<br>LVPLFSK
		AAK49439	Cytotoxin SP15a	3FTx	9	51	24.65	MYMVATPK<br>MYMVATPKVPVKR<br>YVCCNTDR<br>YVCCNTDRCN<br>LVPLFYK<br>CNKLVPLFYK<br>GCIDVCPK
		AAB24494	Vc-5=cytotoxin	3FTx	6.7	43	13.05	YVCCNTDR<br>YVCCNTDRCN<br>GCIDVCPK<br>KLVPLFSK<br>LVPLFSK
		ABU63162	Phospholipase A2 precursor	PLA_2	15.8	6	1.79	NMIQCAGSR
Naja kaouthia; (West Bengal)	F2	P60307	Cytotoxin SP15a	3FTx	6.7	53	19.16	MFMVATPKVPVKR<br>TCPPGKNLCYK<br>RGCIDVCPK<br>GCIDVCPK<br>MFMVATPK
	F4	P86538	Cytotoxin 2a	3FTx	6.7	83	111.74	LVPIASKTCPPGK<br>MFMVSDLTIPVK<br>MFMVSDLTIPVKR<br>NLCYKMFMVSDLTIPVKR<br>LQCNKLVPIASKTCPPGK<br>LQCNKLVPIASK<br>GCIDVCPKNSLLVK
		BAU24674	cytotoxin 15, partial	3FTx	8	51	16.54	MYMVSNKTVPVKR<br>RGCIDVCPK<br>GCIDVCPK<br>GCIDVCPKNSLLVK<br>YVCCNTDRCN
		1H0J	Cardiotoxin A3	3FTx	6.7	65	16.23	MFMVATPK<br>RGCIDVCPK<br>GCIDVCPK<br>LKCNKLVPLFYK<br>YVCCNTDRCN
		Q9I900	RecName: Full=Acidic phospholipase A2	PLA_2	38	5.9	6.29	TYSYECSQGTLTCK<br>CCQVHDNCYGEAEK<br>GGNDACAAAVCDCDR<br>GGSGTPVDDLDR
		BAA36404	Phospholipase A2	PLA_2	45	6.46	6.43	NMIQCTVPSR<br>TYSYECSQGTLTCK<br>CCQVHDNCYNEAEK<br>GGNNACAAAVCDCDR<br>GGSGTPVDDLDR
Naja oxiana (Himachal Pradesh)	F2	4AEA	A Chain A, Long Neurotoxin 1	3FTx	66	8.19	4.99	VDLGCAATCPTVK<br>TWCDAFCSIR<br>RVDLGCAATCPTVK<br>IRCFITPDITSKDCPNGHVCYTK
		BAU24666	cytotoxin 11, partial	3FTx	29	8.41	4.58	MFMVSDLTIPVKR<br>GCIDVCPK
		P25672	RecName: Full=Long neurotoxin 4	3FTx	62	7.47	2.25	VDLGCAATCPTVK<br>TWCDGFCR<br>IRCFITPDITSKDCPNGHVCYTK
		P01415	RecName: Full=Weak toxin CM-2	3FTx	10	5.92	1.7	YLLCCR
		P25668	Long neurotoxin 1	3FTx	52	7.8	9.31	RVDLGCAATCPTVR<br>IRCFITPDITSKDCPNGHVCYTK<br>CFITPDITSKDCPNGHVCYTK<br>CFITPDITSK
		P01400	Weak toxin S4C11	3FTx	37	8.19	7.7	RFYEGNLLGKR<br>LTCLICPEKYCNK<br>LTCLICPEK

								FYEGNLLGKR<br>FYEGNLLGK
		P85520	Oxiana weak toxin	3FTx	43	8.63	2.46	LTCLICPEKYCNK<br>LTCLICPEK<br>YIRGCAATCPEAKPR
		P01401	Weak toxin CM-11	3FTx	42	8.65	2.46	VHTCRNGENQCFKR<br>LTCLICPEKYCNK<br>LTCLICPEK
	F3	AAA90960	cardiotoxin 1e	3FTx	32	8.41	19.64	GCIDVCPK<br>MFMMSDLTIPVK<br>MFMMSDLTIPVKR<br>YVCCNTDRCN
		JAA74657	PLA2-Bra-17	3FTx	8	4.41	2.47	GGSGTPVDDLDR
