# Supplementary figures and images for: Multilevel Comparison of Indian Naja Venoms and Their Cross-Reactivity with Indian Polyvalent Antivenoms
Source: Toxins (Basel). 2023 Apr 1;15(4):258. doi: 10.3390/toxins15040258 (PMC10142961; doi:10.3390/toxins15040258)

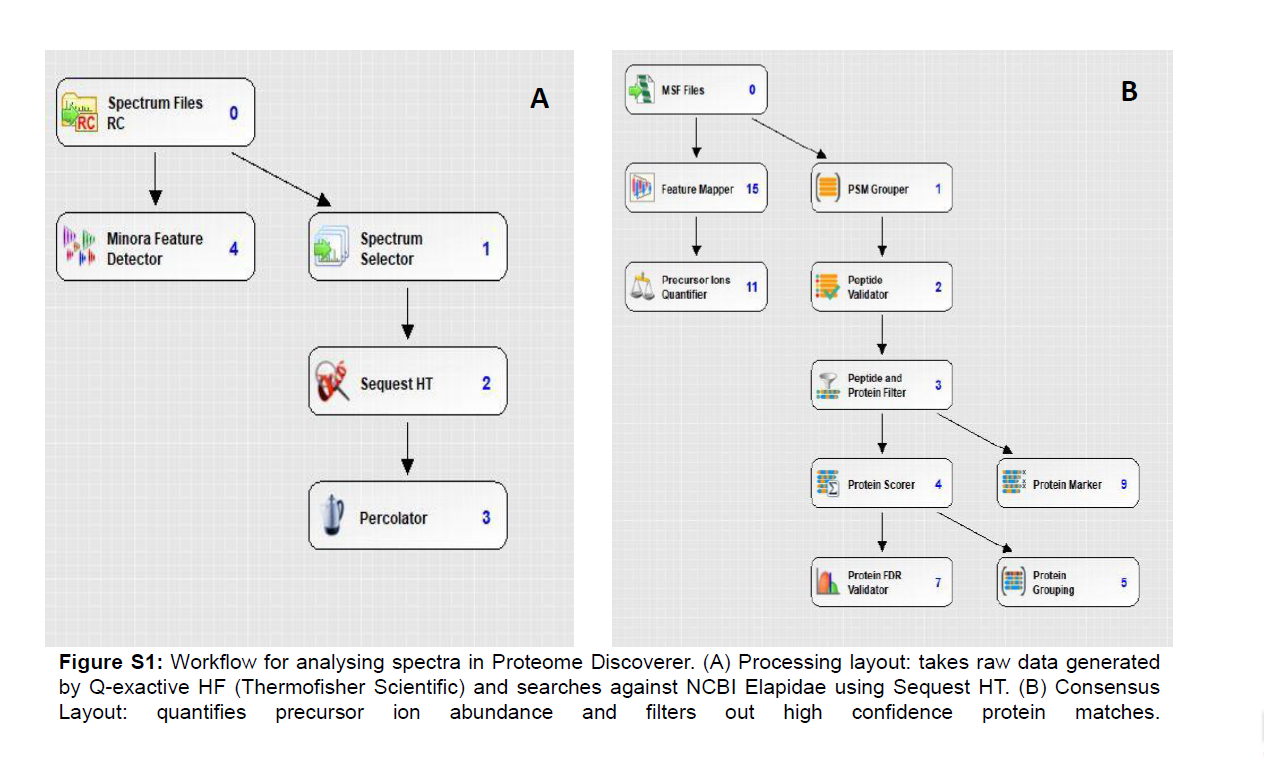

Supplement: Supplementary file 1 [file toxins-15-00258-s001.zip › Supplementary figure S1.png]
